# Effects of perioperative transcutaneous electrical acupoint stimulation on monocytic HLA-DR expression in patients undergoing coronary artery bypass grafting with cardiopulmonary bypass: study protocol for a double-blind randomized controlled trial

**DOI:** 10.1186/s13063-019-3889-z

**Published:** 2019-12-30

**Authors:** Wen-ting Chen, Jin-feng Wei, Lan Wang, Deng-wen Zhang, Wei Tang, Jian Wang, Yue Yong, Jing Wang, Ya-lan Zhou, Lan Yuan, Guo-qiang Fu, Sheng Wang, Jian-gang Song

**Affiliations:** 1 Anesthesiology Department, Shuguang Hospital Affiliated to Shanghai University of Traditional Chinese Medicine, Shanghai, China; 2 Guangdong Cardiovascular Institute & Guangdong General Hospital, Guangdong Academy of Medical Sciences, Guangzhou, Guangdong Province China; 3 Shantou University Medical College, Shantou, Guangdong Province China; 4 Acupuncture and Anesthesia Research Institute, Shuguang Hospital Affiliated to Shanghai University of Traditional Chinese Medicine, Shanghai, China

**Keywords:** CABG, TEAS, Immunosuppression, mHLA-DR

## Abstract

**Background:**

Cardiac surgery involving cardiopulmonary bypass (CPB) is known to be associated with a transient postoperative immunosuppression. When severe and persistent, this immune dysfunction predisposes patients to infectious complications, which contributes to a prolonged stay in the intensive care unit (ICU), and even mortality. Effective prevention and treatment methods are still lacking. Recent studies revealed that acupuncture-related techniques, such as electroacupuncture and transcutaneous electrical acupoint stimulation (TEAS), are able to produce effective cardioprotection and immunomodulation in adult and pediatric patients undergoing cardiac surgery with CPB, which leads to enhanced recovery. However, whether perioperative application of TEAS, a non-invasive technique, is able to improve immunosuppression of the patients with post-cardiosurgical conditions is unknown. Thus, as a preliminary study, the main objective is to evaluate the effects of TEAS on the postoperative expression of monocytic human leukocyte antigen (-D related) (mHLA-DR), a standardized “global” biomarker of injury or sepsis-associated immunosuppression, in patients receiving on-pump coronary artery bypass grafting (CABG).

**Methods:**

This study is a single-center clinical trial. The 88 patients scheduled to receive CABG under CPB will be randomized into two groups: the group receiving TEAS, and the group receiving transcutaneous acupoint pseudo-electric stimulation (Sham TEAS). Expression of mHLA-DR serves as a primary endpoint, and other laboratory parameters (e.g., interleukin [IL]-6, IL-10) and clinical outcomes (e.g., postoperative infectious complications, ICU stay time, and mortality) as the secondary endpoints. In addition, immune indicators, such as high mobility group box 1 protein and regulatory T cells will also be measured.

**Discussion:**

The current study is a preliminary monocentric clinical trial with a non-clinical primary endpoint, expression of mHLA-DR, aiming at determining whether perioperative application of TEAS has a potential to reverse CABG-associated immunosuppression. Although the immediate clinical impact of this study is limited, its results would inform further large-sample clinical trials using relevant patient-centered clinical outcomes as primary endpoints.

**Trial registration:**

ClinicalTrials.gov, NCT02933996. Registered on 13 October 2016.

## Background

Cardiac surgery involving cardiopulmonary bypass (CPB) is known to be associated with immune dysfunctions characterized by initial pro-inflammatory response and subsequent anti-inflammatory response [1]. If the following anti-inflammatory response is persistent and severe, it will lead to a long-lasting immunosuppressive state. This may result in an increased susceptibility to postoperative infectious complications, such as pneumonia or impaired wound healing, and thus a prolonged stay in the intensive care unit (ICU), or even mortality [2–5].

During CPB surgery, the exposure of blood to the non-physiological surfaces of the CPB apparatus [6], cardiac arrest and ischemia/reperfusion injury of organs [7], and translocation of endotoxins (lipopolysaccharide, LPS) across the ischemic gut wall [8–10] trigger a pronounced pro-inflammatory response, which is characterized by circulating cytokines, activation of endothelial cells and neutrophils, complement activation, circulating arachidonic acid metabolites, platelet-activating factors, and endothelins [11, 12]. As a physiologic countermeasure to ameliorate this harmful overactivation of innate immunity, a compensatory anti-inflammatory response often follows, which is known to cause a transient immunosuppression. It comprises secretion of anti-inflammatory cytokines such as interleukin (IL)-10 by monocytes and T cells, down-regulation of inflammatory cell surface receptors on neutrophils, impaired monocytic response to bacterial endotoxins [13, 14], reduced production of interferon-γ (IFNγ), IL-2, and tumor necrosis factor (TNF)-α, as well as peripheral blood mononuclear cell (PBMC) proliferation in response to stimulation with phytohemagglutinin [15]. Generally, this immunosuppression is temporary and can be restored to normal. However, when serious and persistent, this immune imbalance is considered to contribute to postoperative infectious complications. Unfortunately, the counterbalancing long-lasting immunosuppressive responses still remain a clinical challenge.

Acupuncture is an ancient, non-drug treatment technology originating in China, which has been widely used worldwide. Recently, more and more studies have revealed that acupuncture is able to effectively regulate the function of the immune system, and this technique has been clinically regarded as a primary or adjuvant therapy measure for some immune-related diseases (e.g., asthma, allergic rhinitis, and rheumatic arthritis) [16–18]. In recent years, acupuncture-related techniques began to be applied to cardiac surgery for better recovery. Yang et al. [19] have shown that for adult patients undergoing heart valve replacements, electroacupuncture (EA) pretreatment can alleviate cardiac ischemia-reperfusion injury indicated by reduced overall serum troponin I release and dosage of inotropic drug use after surgery. Also, the ICU stay time can be shortened. Subsequently, pretreatment of transcutaneous electric acupoint stimulation (TEAS), a non-invasive acupoint stimulation technique, has produced similar cardioprotective effects in pediatric patients undergoing cardiac surgery. Moreover, alleviated inflammation indicated by reduced C-reactive protein (CRP) level in the early postoperative period was observed [20].

Experimental studies also demonstrate that EA at the Zusanli (ST36) acupoint suppresses surgical trauma stress-induced lymphocyte apoptosis [21] and increases lymphocyte proliferation and IL-2 production in surgically traumatized rats [22–24] and IFNγ production of the spleen in mice [25]. Wang et al. further indicated that EA administration after surgical trauma increased Th1 cytokine protein and mRNA expression (IL-2 and IFNγ), and suppressed Th2 cytokine protein and mRNA expression (IL-4 and IL-10), involving the signaling pathways of ERK1/2, p38, NF-κB, and AP-1 in rats. These findings suggest that EA may improve immune suppression after surgical trauma [26].

TEAS involves no risk of infection, needle-induced contagious disease, or fear of stimulation and is more “user friendly” with minimal training, which is more convenient for clinical application [27]. However, whether perioperative application of TEAS is able to improve postoperative immunosuppression of patients receiving on-pump CABG is unknown.

In the current study, we will attempt to evaluate potential TEAS-induced reversal of CABG-associated immunosuppression. Previous clinical studies have shown that whether the patient is in a conscious state (i.e., preoperative or postoperative) or an anesthetic state, TEAS or EA applied to various surgical operations can produce beneficial effects, such as preventing hyperglycemia [28], reducing intraoperative opioid consumption [29], relieving post-hemorrhoidectomy-associated pain [30], and improving immune and stress responses to surgery [31]. In order to maximize the possible benefit of TEAS, the perioperative administration (i.e., preoperative, intraoperative, and postoperative) of TEAS will be chosen in our trial.

Considering that this is a preliminary monocentric study, monocytic human leukocyte antigen (-D related) (mHLA-DR), a standardized “global” biomarker of injury- or sepsis-associated immunosuppression, serves as a primary endpoint, and other laboratory parameters (e.g., IL-6, IL-10) and clinical outcomes (e.g., postoperative infectious complications, ICU stay time, and mortality) as the secondary endpoints. Monocytic HLA-DR is a major histocompatibility complex (MHC) class II molecule and is predominantly expressed on monocytes/macrophages [32]. Its surface expression is indispensable for antigen presentation [32]. Increased mHLA-DR expression reflects activation of immune cells, while diminished expression exhibits a phenotype with down-regulation of antigen-presenting capacity and a shift from pro- to anti-inflammatory cytokine production [33, 34]. Moreover, surface expression of mHLA-DR is crucial for induction of adaptive immune responses [32, 35]. More importantly, accumulated clinical evidence has indicated that its persisting decreased expression is associated with adverse clinical outcomes (e.g., secondary infection risk, mortality) in patients with trauma [36], burns [37], pancreatitis, [38, 39] solid organ transplantation [40], hepatic [41] or renal injury [42], stroke [43], myocardial infarction/heart failure and cardiac arrest [44–47], as well as sepsis [48]. The same is true of cardiac surgery with CPB. The quantification of mHLA-DR expression shows the best predictive power on outcome in pediatric and adult patients. Postoperative reduced mHLA-DR was associated with increased length of ICU stay/mechanical ventilation and development of postoperative sepsis [49, 50].

In addition, some immune indicators, such as high mobility group box 1 protein (HMGB1) and regulatory T cells (Treg), possibly related to the mechanism of TEAS would also be measured. HMGB1, originally described as a DNA-binding protein and passively released by necrotic cells and actively released by macrophages/monocytes, was discovered to be an essential cytokine that mediates the response to infection, injury, and inflammation [51]. Our previous animal study has shown that EA can inhibit excessive release of HMGB1 following myocardial ischemia and attenuate the associated inflammatory responses and myocardial injury during reperfusion [52]. Recently, HMGB1 was found to directly enhance the immune inhibitory functions of Treg and limit the number and activity of conventional T cells [53, 54].

Treg cells are responsible for limiting tissue damage and inflammation associated with both innate and adaptive immune responses [55]. However, overactivation of Treg contributes to immunosuppression [56]. It was shown that the population of Treg in PBMCs was significantly increased at 48 h and 96 h after CABG with CPB [57], which may contribute to CABG-associated immunosuppression. Thus, we speculate that the potential inhibition of excessive HMGB1 release of TEAS, consequently leading to attenuated function of Treg, may be associated with TEAS-induced reversal of immunosuppression, characterized by increased mHLA-DR.

### Objective

The primary objective of the study is to evaluate the effects of perioperative TEAS on mHLA-DR expression in patients undergoing CABG with CPB.

## Methods/design

### Study design

**Fig. 1:**
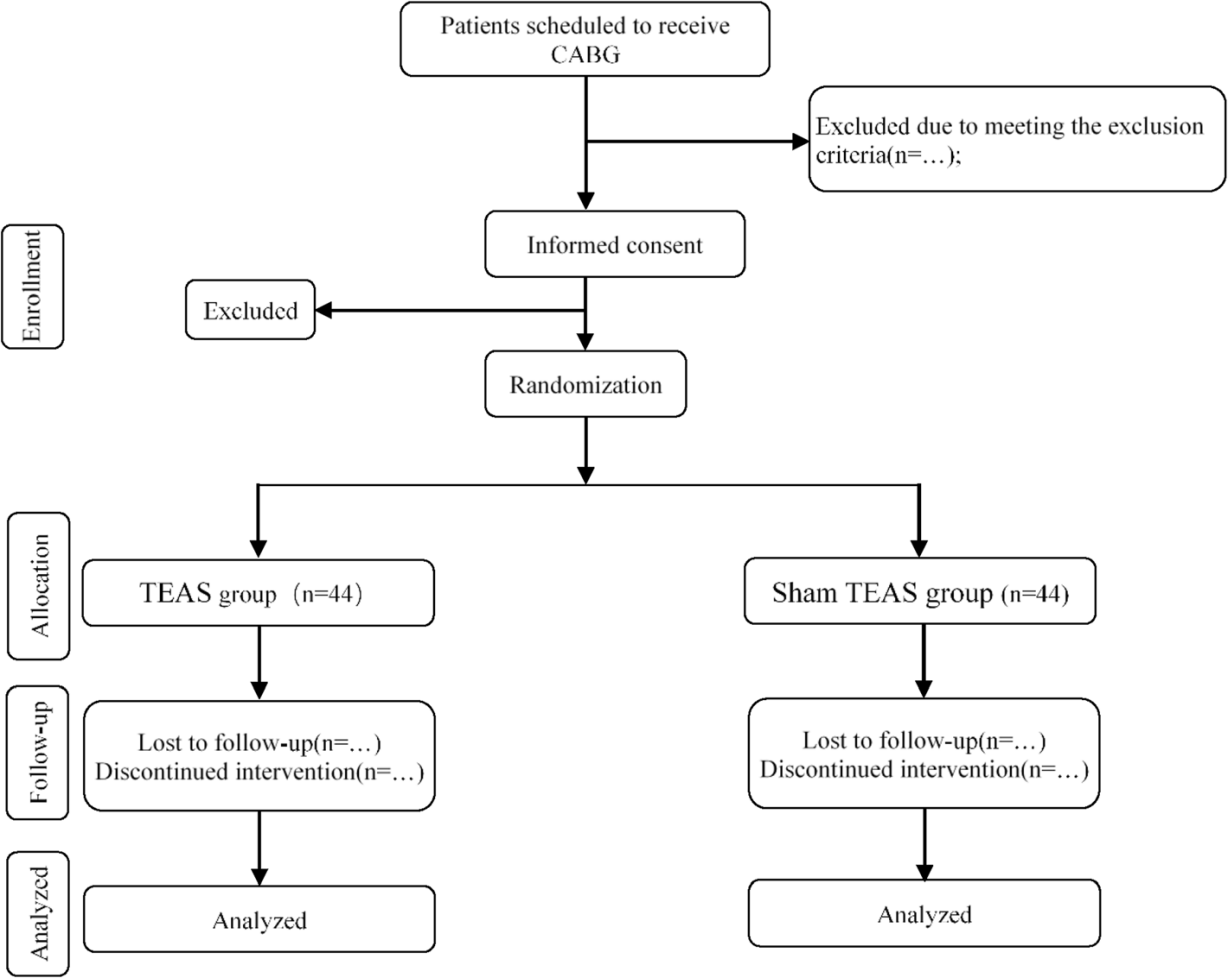
Flowchart of the study

This study is a preliminary, single-center, double-blind, randomized and controlled clinical trial (number of samples, *n* = 88) to explore the effects of TEAS therapy on improvement of postoperative immunosuppression indicated by diminished HLA-DR expression of patients receiving CABG (Fig. 1). The trial will commence after ethical approval has been obtained from the Ethics Committee of Shuguang Hospital Affiliated to Shanghai University of Traditional Chinese Medicine. All study-related procedures will be performed only after subjects have given their written informed consent. The trial is designed following the Consolidated Standards of Reporting Trials (CONSORT) guidelines, the Standard Protocol Items: Recommendations for Interventional Trials (SPIRIT) checklist (Additional file 1), and Standards for Reporting Interventions in Controlled Trials of Acupuncture (STRICTA) recommendations.

### Participants

#### Current sample size justification

We calculated the sample size from the pilot study in our hospital based on the primary outcome: monocytic human leukocyte antigen DR (mHLA-DR). In that study, the expression of mHLA-DR 3 days after surgery was 36.17 ± 5.42% in the TEAS group and 27.33 ± 3.50% in the Sham TEAS group, with a power of 80% and a 5% type I error rate. Assuming that the dropout rate is 10%, 88 patients (*n* = 44 for each group) are needed.

#### Subject recruitment

Patients will be recruited from the Department of Thoracic and Cardiovascular Surgery, at Shuguang Hospital Affiliated to Shanghai University of Traditional Chinese Medicine. Potentially eligible subjects who are scheduled for CABG under CPB for coronary disease will be invited to participate. Patients will be referred from a cardiothoracic surgeon, and then a research assistant will approach the subjects in the general ward pre operation. Patients will then be screened and consented for the study. Following the consent, eligible participants will be block randomized into two groups: (1) TEAS group (*n* = 44) and (2) Sham TEAS group (*n* = 44). All patients will receive standard operative procedure and postoperative analgesia management. The patients of the TEAS group will receive TEAS therapy in the perioperative period, and the Sham TEAS group will receive “pseudotherapy” performed without electrical stimulation in the perioperative period. Assessments will be conducted during the perioperative period until 30 days after surgery.

#### Inclusion criteria

The inclusion criteria are as follows:

1. Aged 18–75 years, male and female
2. Patients diagnosed with coronary disease and scheduled to receive CABG
3. Body mass index (BMI) measure of 18.5 kg/m^2^ < BMI ≤ 30 kg/m^2^
4. Patients with Grade of I–III according to American Society of Anesthesiologists (ASA)
5. Patients first receiving CABG under extracorporeal circulation.

#### Exclusion criteria

The exclusion criteria are as follows:

1. Presence of surgical incision or scar at Zusanli acupoint (ST36)/Shenshu (BL23) acupoint
2. Patients with local skin infection at acupoint
3. Patients with nerve injury on upper or lower limbs
4. Patients with history of spinal surgery
5. Patients who have participated in another clinical trial in the last 4 weeks
6. Patients using a pacemaker
7. Patients who have pain before surgery who are using a central analgesic drug or those who are drug abusers (e.g., opioids) or dependent users
8. Patients who have severe central nervous system disease or severe mental disease
9. Patients with an alcoholic history
10. Patients who have received emergency coronary bypass operation due to acute myocardial infarction.

### Randomization and blinding

A computer generates a random number sequence. The allocations will be printed and placed in separate sealed envelopes. The patients will be randomly divided into either the TEAS group or the Sham TEAS group in a 1:1 ratio. We set a blind code in case patients have adverse effects. The random code and blind code will be conducted using opaque envelopes by a “third party” independent of the study. The envelopes will be sealed and shuffled, and the assignment records will not be disclosed until the end of the study. Trial participants, cardiothoracic surgeons, anesthesiologists, outcome assessors, and data analysts will be blinded to the treatment allocation to minimize potential sources of bias. Only the nurse of the anesthesiology department (having received specialized acupuncture training) will know the participants’ group allocations. However, this nurse will not know any other information about the patients.

### Interventions

**Fig. 2:**
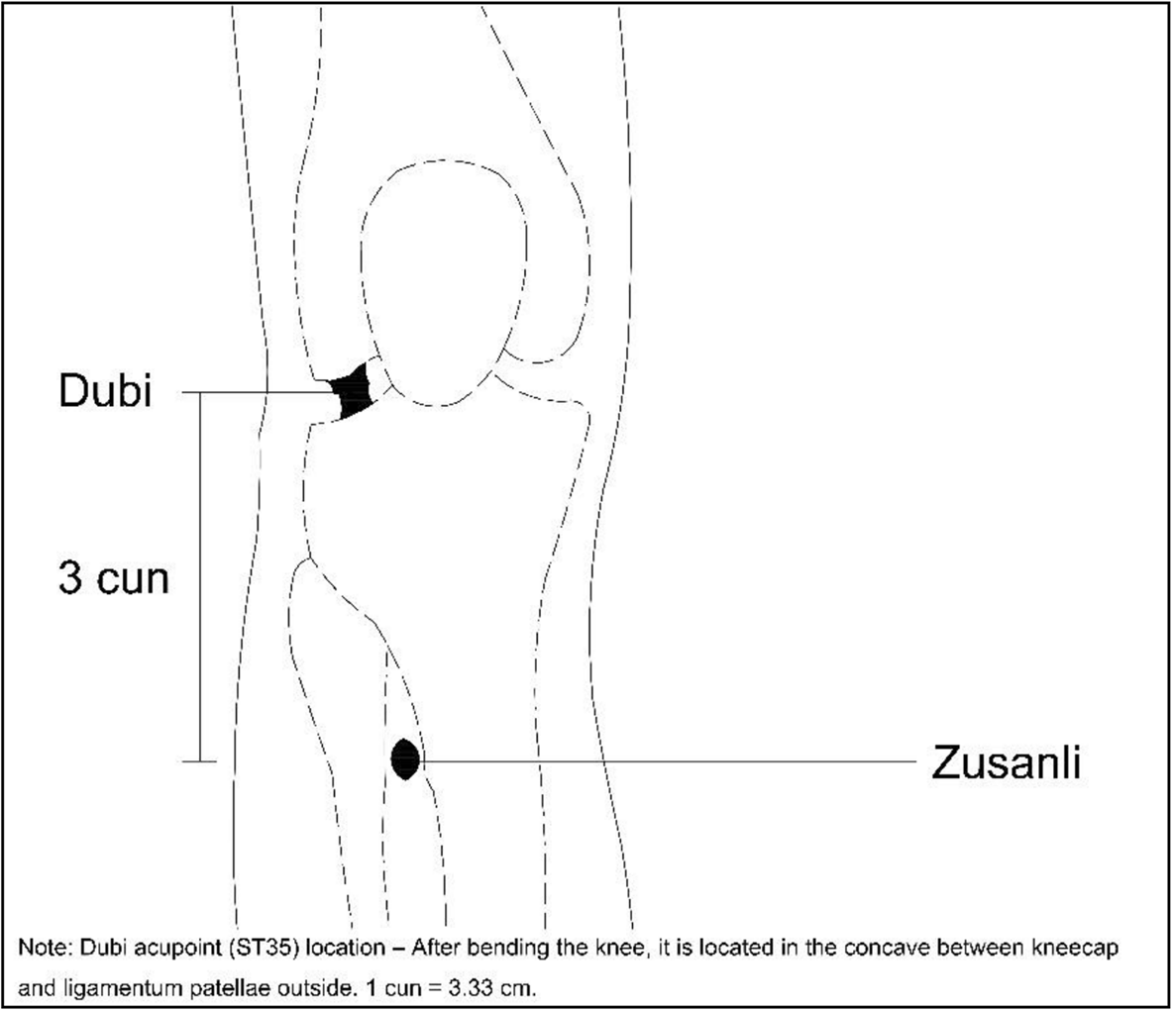
Zusanli location: outside of the shank, 3 cun(10 cm) below Dubi acupoint and a finger’s width (middle finger) to tibial front edge

**Fig. 3:**
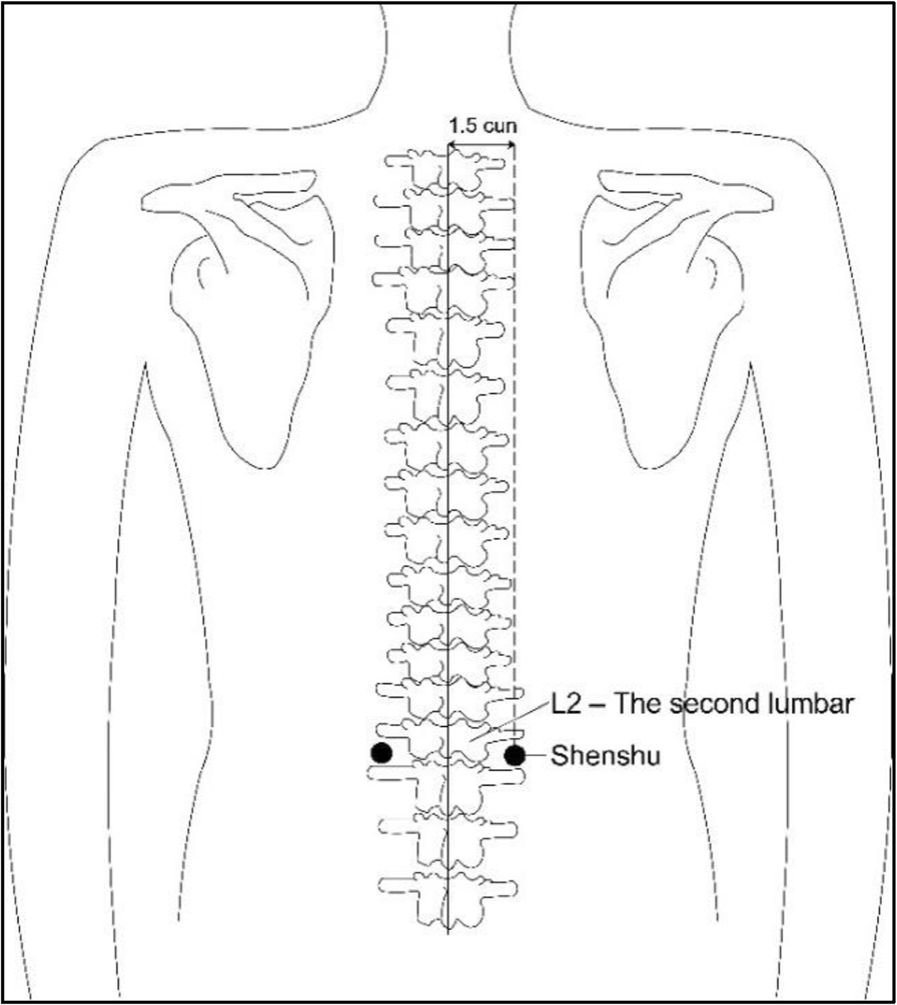
Shenshu location: below the spinous process of the second lumbar vertebra, 1.5 cun (5 cm) to the central line

**Fig. 4:**
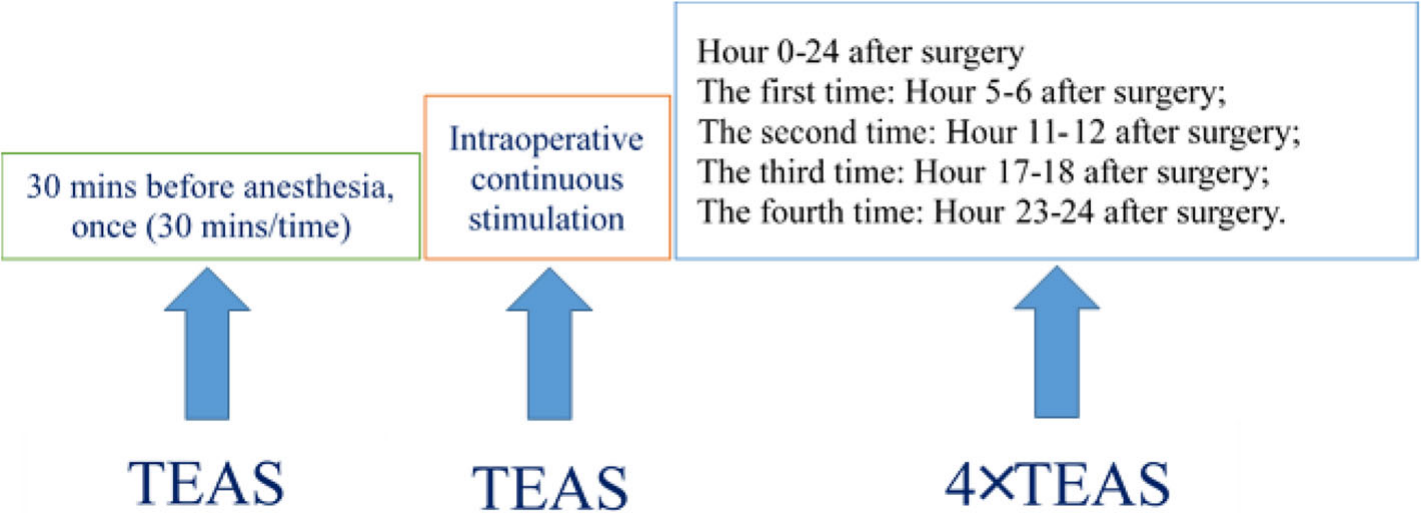
The patients will receive TEAS therapy 30 min before anesthesia (one stimulation for 30 min), during the period of surgery (stimulation for the whole course) and within 24h after surgery (four times of stimulation, each for 30min)

**Fig. 5:**
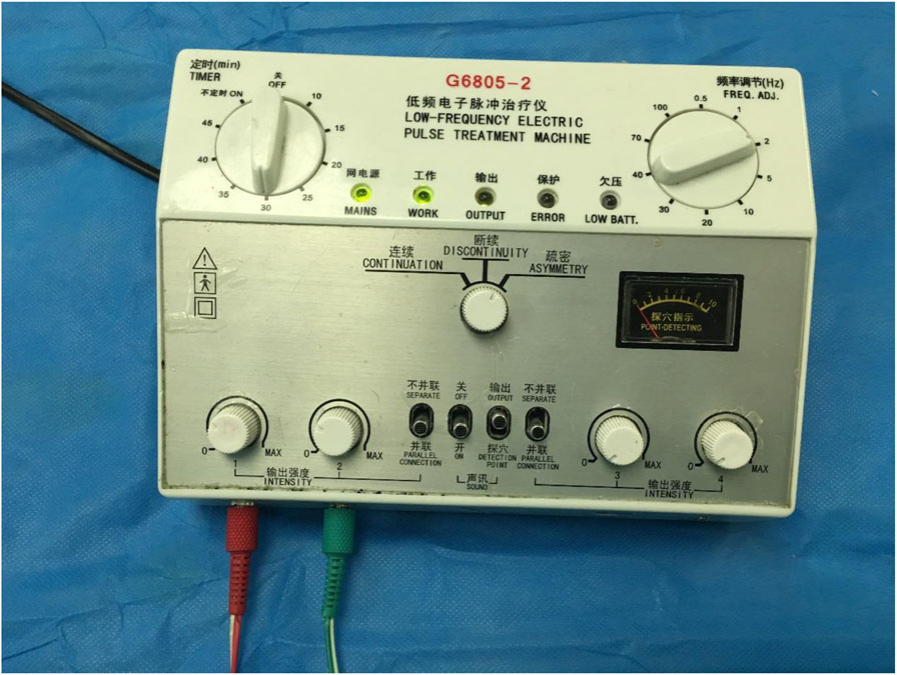
We will use Low-frequency electronic pulse therapeutic device G6805-2 (Huayi, Shanghai, China) for TEAS therapy, the frequency will be 2/100 Hz alternating and the intensity will be 15mA

**Fig. 6:**
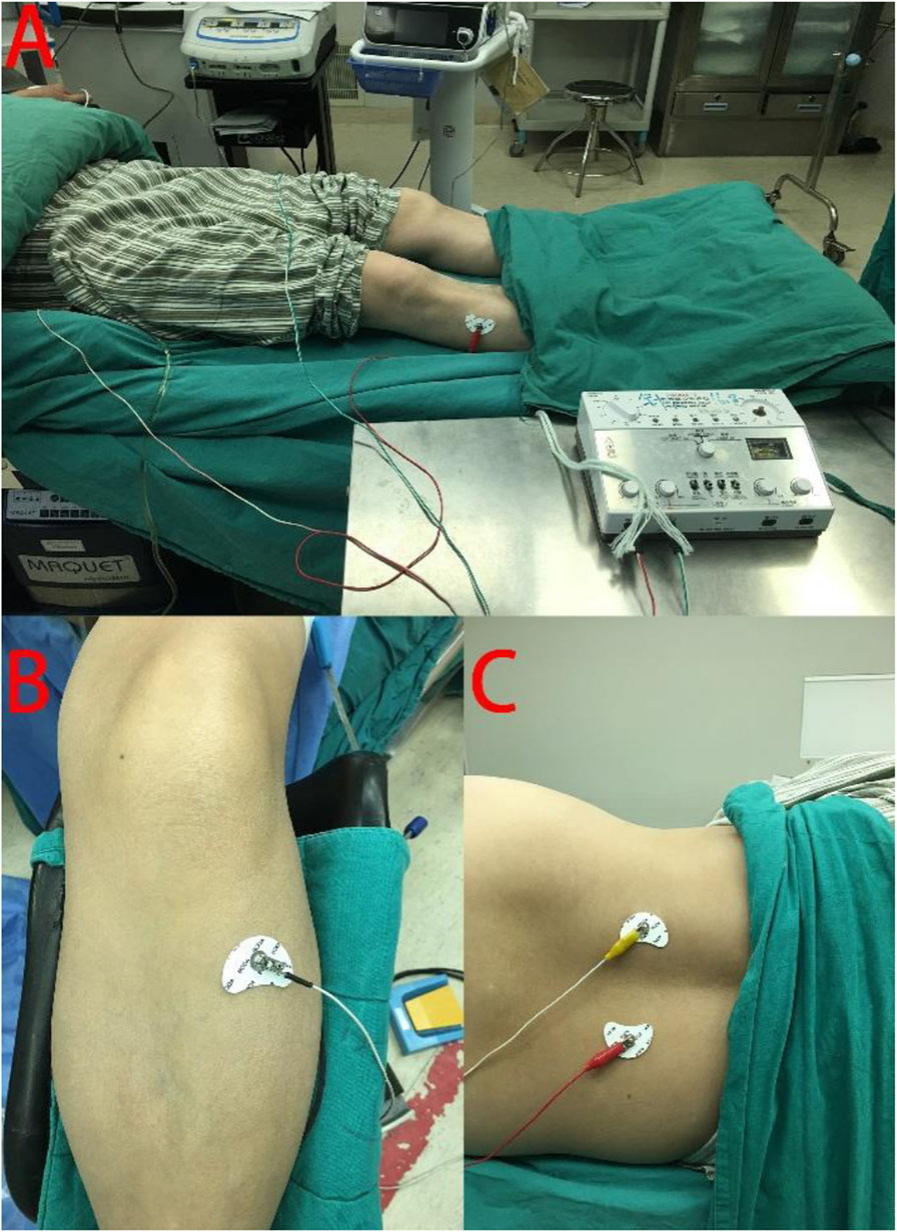
**a**: Patient received TEAS therapy at bilateral Zusanli acupoint (ST36) and Shenshu acupoint (BL23) during the perioperative period. **b**: Zusanli acupoint (ST36) is identified. **c**: Shenshu acupoint (BL23) is identified

This trial will include two groups: the TEAS group and the group receiving transcutaneous acupoint pseudoelectric stimulation (Sham TEAS). In each group, there will be 44 patients receiving CABG with CPB. The patients of the TEAS group will receive TEAS therapy in the perioperative period, and in the Sham TEAS group, pseudotherapy without actual electrical stimulation will be performed in the perioperative period. The intervention details are as follows:

1. Selection of acupoints: Zusanli acupoint (ST36), Shenshu acupoint (BL23) (see Figs. 2 and 3):
  - Zusanli location: outside of the shank, 3 cun (10 cm) below Dubi acupoint (ST35) and a finger’s width (middle finger) to tibial front edge
  - Shenshu location: below the spinous process of the second lumbar vertebra, 1.5 cun (5 cm) to the central line
2. Stimulation timing: before anesthesia plus intraoperative plus postoperative (see Fig. 4):
  - 30 min before anesthesia: one stimulation for 30 min
  - Intraoperative: stimulation for the whole course
  - Postoperative: 0–24 h: four times of stimulation (30 min per stimulation). 5–6 h after the surgery (the first time), 11–12 h after the surgery (the second time), 17–18 h after the surgery (the third time), 23–24 h after the surgery (the fourth time)
3. TEAS parameters
  - Frequency: 2/100 Hz alternating
  - Intensity: 15 mA
  - Low-frequency electronic pulse therapeutic device G6805-2 (Huayi, Shanghai, China) (see Fig. 5)
4. Current intensity: main difference between the study group and control group
  - TEAS group: the acupoints, including Zusanli and Shenshu, are identified before electrical stimulation with surface electrodes (Fig. 6). Selection of these acupoints was based on a consensus between the acupuncturists of the study.
  - Sham TEAS group: No electrical stimulation is actually performed in the Sham TEAS group. In the Sham TEAS group, pseudostimulation is provided by deliberately connecting the electrodes to the incorrect output socket of the EA device; thus, there is no flow of electric current. Patients can see the output light flashing, but no current is transmitted throughout the procedure. Patients would be told that the stimulation frequency selected was not perceivable by humans.
5. Anesthesia protocol
  - Medication before the anesthesia: morphine 0.1 mg/kg
  - Anesthesia induction
    1. Sufentanil 0.3–0.5 μg/kg
    2. Propofol, target-controlled infusion (TCI): 2.0–3.5 μg/ml
    3. Dextromidine 0.5–1.0 μg/kg/h or midazolam 0.05–0.1 mg/kg
    4. Lidocaine 1 mg/kg (maximum dose not higher than 50 mg)
    5. Rocuronium bromide 0.9–1.2 mg/kg
  - Maintenance of anesthesia
    1. Narcotic analgesics: common sufentanil by 0.2–0.5 μg/kg by times (intravenously) or remifentanil by 0.05–0.2 μg/kg/min continuous intravenous pump injection, addition of sufentanil by 10–20 μg before skin incision and sternum splitting.
    2. Inhaled general anesthetics: sevoflurane and isoflurane can be inhaled discontinuously as requested with minimum alveolar concentration (MAC) 0.7–1.0.
    3. Muscle relaxant: common vecuronium bromide and rocuronium bromide, etc.
    4. After the completion of tracheal intubation, the anesthesia machine is connected immediately, and end-tidal carbon dioxide (ETCO_2_) is examined, and the breathing sound of both lungs is auscultated to determine the position of endotracheal tube.
    5. Common parameters of mechanical ventilation: tidal volume (VT) 7–8 ml/kg, respiratory rate (RR) 10–12 bpm, arterial partial pressure of oxygen (PaO_2_) 200 mmHg, arterial partial pressure of carbon dioxide (PaCO_2_) 35–45 mmHg, fraction of inspired oxygen (FiO_2_) 80%.

### Outcomes

#### Primary outcome measures

With mHLA-DR as the primary outcome of this clinical trial, the improvement of postoperative immunosuppression will be evaluated.

Peripheral blood is collected from the patient to test this indicator at the following time points: one day before surgery, Day 1 after surgery (one day after surgery), Day 3 after surgery (3 days after surgery), and Day 5 after surgery (5 days after surgery).

The percentage of HLA-DR^+^/CD14^+^ cells in all CD14^+^ cells will be determined by flow cytometry (Becton-Dickinson, Franklin Lakes, NJ, USA) in the central lab of Shuguang Hospital Affiliated to Shanghai University of Traditional Chinese Medicine.

#### Secondary outcome measures

The secondary outcome measures are as follows:

1. Related indicators of immunosuppression include interleukin (IL)-6, IL-10, C-reactive protein (CRP), postoperative infectious complications (pneumonia, incision infection, and indwelling catheter infection), ICU stay time, and mortality. The examination methods and time points are as follows:
  - IL-6 and IL-10: Plasma levels of IL-6 and IL-10 will be determined using the ELISA (enzyme-linked immunosorbent assay) with high sensitivity kits (ABCAM, Shanghai, China) one day before surgery and on Days 1, 3, and 5 after surgery.
  - CRP: CRP expression level in blood samples, determined with automatic biochemical analyzer (Beckman Coulter, Atlanta, GA, USA), one day before surgery and on Days 1, 3, and 5 after surgery.
  - Postoperative infectious complications:
    1. Pneumonia: Pneumonia was defined according to the Centers of Disease Control and Prevention (CDC) guidelines [58]. Postoperative healthcare-associated pneumonia will be assessed throughout by radiograph suspicious for pneumonia, clinical signs and symptoms suspicious for pneumonia and positive culture from bronchoalveolar lavage, a positive blood culture not related to another infection, or a positive sputum culture.
    2. Postoperative infection other than pneumonia: observe the incidence of incision infection and indwelling catheter infection after the surgery (fever, bacterial culture).
      We will record postoperative infectious complications according to the patient’s medical history, imaging examination, clinical signs, and sputum culture within 30 days after the operation.
  - ICU stay time: We will record the length of ICU stay after surgery (days [*d*], mean ± standard deviation [SD]).
  - Mortality: We will record a 30-day mortality rate after surgery.
2. The indicators of related mechanisms studied include high mobility group box 1 proteins (HMGB1), regulatory T cells (Treg), and CD4^+^ T cells. The examination methods and time points are as follows:
  - HMGB1: HMGB1expression level in blood samples, ELISA kit (ABCAM, Shanghai, China) one day before surgery, and on Days 1, 3, and 5 after surgery
  - Treg: Percentage of CD4^+^/CD25^+^ T cells in CD4^+^ T cells, flow cytometry (Becton-Dickinson, Franklin Lakes, NJ, USA), one day before surgery and on Days 1, 3, and 5 after surgery
  - CD4+ T cells: CD4+ T cell number/ml blood, flow cytometry (Becton-Dickinson), one day before surgery and on Days 1, 3, and 5 after surgery.

All the related indicators and the indicators of related mechanisms will be tested in the central lab of Shuguang Hospital Affiliated to Shanghai University of Traditional Chinese Medicine.

### Statistical analysis

All statistical analyses of the data will be performed using SPSS program V.21.0 (SPSS Inc., Chicago, IL, USA). A *P* value < 0.05 will be considered statistically significant. The intention-to-treat (ITT) approach will be used. Measured data will be expressed as mean ± SD (^−^*x*± *s*) if it obeys a normal distribution or an approximate normal distribution. The median (interquartile range [IQR]) will be used if the data do not obey the normal distribution and count data will be expressed in terms of the number of cases.

To analyze the primary outcome, the expression of mHLA-DR (the percentage of HLA-DR+/CD14+ cells in all CD14+ cells) will be calculated by repeated measures analysis of variance (ANOVA), and the comparisons between the two groups will be made using the Student’s *t* test. For the secondary outcomes, we will use chi-square tests for categorical data and repeated measures ANOVA or the Wilcoxon rank sum test for continuous data, according to whether the data are normally distributed. The variance analysis will be performed for the difference between the two groups and within a group. A stratified analysis will be performed to control the confounding factor if necessary. Data analysis will be conducted by statisticians who are independent of the research team.

### Data collection and management

The data will be collected as primary and secondary outcome measures, with the above-described method. All data will be saved safely on an internal server of Shuguang Hospital, with complete confidentiality. The participants of this study will be cited with a code different from their real names. The data management program will be approved by the trial manager and other clinicians before the registration of the first participant.

### Adverse events

The status related to adverse events is acquired according to the self-report of the patient or direct observation of clinicians or by non-induced query of the patient, and his/her clinical safety will be evaluated (see Table 1).

**Table 1:** Clinical safety evaluation in perioperative period: any of the listed conditions is considered a *complication of the perioperative period*

1	Postoperative arrhythmia	Postoperative atrial fibrillation, atrial flutter, supraventricular tachycardia, ventricular tachycardia, ventricular fibrillation, ventricular flutter, cardiac arrest, atrioventricular block of 2^nd^ degree or above, frequent atrial premature beat and ventricular premature beat significantly affecting the stability of hemodynamics (indicated in electrocardiogram [ECG])		
2	Pneumonia	Body temperature above 38.5 °C (indicated in chest film)		
3	Acute lung injury	(1) Acute onset, with pathogenic factors		
		(2) Oxygenation index (arterial partial pressure of oxygen/fraction of inspired oxygen, PaO_2_/Fi0_2_) < 300 mmHg (1 mmHg = 0.133 kPa) not referring to positive end-expiratory pressure (PEEP) level		
		(3) Frontal X-ray chest film revealed patchy shadows in both lungs		
		(4) Pulmonary artery incarceration pressure < 18 mmHg or no clinical evidence of increased pressure in left atrium		
		(5) Acute paroxysmal respiratory failure		
4	Pulmonary atelectasis	Indicated in chest film		
5	Intraoperative and postoperative myocardial infarction	Manifestation of myocardial infarction symptoms or change of ECG ST segment, continuous increase of myocardial enzyme, especially cardiac troponin I (cTnI), accompanied with dynamic change of ST segment		
6	Postoperative cardiac insufficiency	The postoperative cardiac output (CO) is lower than lower limit of normal value or there are symptoms and vital signs of heart failure	(1) Left cardiac insufficiency	*Symptoms*: dyspnea; coughing, expectoration, and hemoptysis; cyanosis, fatigue, and weakness
				*Vital signs*: expansion of border of cardiac dullness, left lower shifting of cardiac impulse with elevating sensation. Accelerated heart rate, diastolic gallop heard in apex, alternative pulse in severe case. Moist and dry rales are heard in the bottom of both lungs. Wheezing rale and dry rales may be accompanied with secondary bronchial spasm
			(2) Right cardiac insufficiency	*Symptoms*: reduced urine volume, increased nocturnal enuresis, swelling pain in liver region or even occurrence of jaundice; inappetence, dyspepsia, nausea, vomiting and diarrhea
				*Vital signs*: expansion of border of cardiac dullness, apex beating showing elevating sensation, diffuse beating range, accelerated heart rate
				Distention of jugular vein, liver swelling with tenderness, hepatojugular reflux sign positive; pitting edema, right heart failure
				Typical vital signs of failure, mostly in the body drooping part
			(3) Whole cardiac insufficiency	Coexistence of clinical manifestations of left and right cardiac insufficiency, but principally one of them
7	Postoperative respiratory insufficiency	Patients showing intracardiac anatomical shunt and cardiac volume decrease when they breath in indoor air at static conditions will be excluded; arterial partial pressure of oxygen (PaO_2_) is lower than 8 kPa (60 mmHg) or accompanied with partial pressure of carbon dioxide (PaCO_2_) higher than 6.65 kPa (50 mmHg)		
8	Postoperative hemorrhage of digestive tract	Ulcer bleeding or bloody gastric content caused by mucosal ischemia of gastrointestinal tract, hematemesis, tarry stool or hemafecia		
9	Postoperative hepatic insufficiency	Severe hepatocellular damage, causing significant metabolism, secretion, synthesis, biotransformation, and immune function disorder, clinical syndrome of edema in the organism, jaundice, hemorrhage, infection, renal function disorder, and hepatic encephalopathy, etc.		
	Postoperative renal insufficiency	Rapid decrease of renal excretory function in short term, and daily mean increase of serum creatinine ≥ 44.2 μmol/L and exacerbation of existing renal insufficiency		
10	Postoperative infection other than lung infection	Including hematogenous infection, infections of digestive tract, urinary system, wound, skin, and indwelling catheter		
11	Postoperative cerebral ischemia and hypoxic disease	Including cerebral infarction, cerebral thrombosis, cerebral hemorrhage, transient cerebral ischemic attack, and diffuse cerebral ischemia and hypoxic disease		
12	Prolongation of postoperative hospital stay	Postoperative hospital stay exceeds 14 days		
13	Acute kidney injury	(1) Increase of plasma creatinine within 48 h ≥ 0.3 mg/dL (≥ 26.5 μmol/L)		
		(2) Plasma creatinine within 7 days ≥1.5 times the basic value		
		(3) Urine volume within 6 h lower than 0.5 ml/kg/h		
14	Death in perioperative period: definition	(1) Death within 30 days after surgery		
		(2) Death in hospital stay after surgery		
		(3) Death caused by surgical reasons after discharge		

### Quality control

The chief surgeon of the thoracic surgery department, the anesthetist to implement anesthetic management, the nurse of the anesthesiology department to carry out TEAS (having received specialized acupuncture training) as well as blood sampling personnel of clinical lab and data recording personnel are fixed to avoid bias from human operations. Specialized acupuncture training mainly includes selection of acupoints and the TEAS operation standard and procedure (see Table 2).

**Table 2:** TEAS operation standard and procedure

1	Determination of position	The patient takes a supine position
2	Inspection of equipment	Confirm normal operation of electric acupuncture apparatus
3	Area and acupoint locating	The Zusanli acupoint and Shenshu acupoint are determined by feeling and pressing the point for acupuncture
4	Local skin preparation	Prepare the skin at the acupuncture point, disinfect from the center with 75% ethanol cotton ball in circle to wipe off the sebum
5	Selection of surface electrodes	Select surface electrodes specially used for TEAS
6	Acupoint patching	Attach the electrode slices specially used for TEAS on the acupoints, press to confirm they are securely attached
7	Connection of electrode slices to equipment	Connect Zusanli acupoint and Shenshu acupoint on one side to 2 electrodes of the same wire, and those of the other side to another 2 electrodes of the same wire; both wires are connected to the same electric acupuncture apparatus
8	Acupoint electric stimulation	Confirm the electric acupuncture apparatus is in power-up state, turn on the electric acupuncture apparatus, select corresponding parameters, and initiate TEAS therapy according to the patient’s tolerance to electric stimulation
9	Maintenance treatment	Maintain electric stimulation for 30 min, instruct the patient to protect the surgery area in acupuncture pin setting process, and closely examine the patient for adverse reactions of fainting, vomiting, and pain during acupuncture treatment; provide symptomatic treatment if needed
10	End of treatment	Turn off the electric acupuncture apparatus, remove the electrode slices, and clear away connection wires. Check redness and swelling or other injury on the skin where electrode slice was attached, and provide symptomatic treatment if these symptoms occur

## Discussion

Patients undergoing CABG with CPB are more prone to have immunosuppression, which may lead to postoperative infectious complications and a prolonged ICU stay, and even mortality. Previous studies have proved that acupuncture was able to improve patients’ immune function. TEAS has a similar efficacy as electroacupuncture, but it is easier to operate, non-invasive, and easy to accept by patients. The current study aims to evaluate potential TEAS-induced reversal of decreased mHLA-DR (a standardized “global” biomarker of immunosuppression) and explore the possible underlying mechanism related to TEAS.

Although the immediate clinical impact of this study is limited, its results will inform further research. Following demonstration of possible immunological efficiency, biomarker-guided immunological interventions of TEAS for immunosuppression should be performed in populations with sufficiently large sample size using relevant patient-centered clinical outcomes (e.g., mortality or infectious complications). Only in this way can we finally determine the clinical value of TEAS for counterbalancing CABG-associated immunosuppression and promote its application.

## Trial status

This is the third protocol version, updated on October 31, 2019. The recruitment began on December 1, 2017 and should be completed by December 31, 2019. At the time of manuscript submission, the study was in recruitment phase.

## Supplementary information

**Additional file 1.** SPIRIT 2013 checklist: recommended items to address in a clinical trial protocol and related documents.

## Data Availability

The final trial dataset will only be accessible to the study investigators.

## Abbreviations

BMI: Body mass index
CABG: Coronary artery bypass grafting
CD4^+^ T cell: CD4 positive thymocyte cell
CPB: Cardiopulmonary bypass
CRP: C-reactive protein
FiO_2_: Fraction of inspired oxygen
GA: General anesthesia
HMGB1: High mobility group box 1 protein
IL-6: Interleukin 6
ITT: Intention-to-treat
mHLA-DR: Monocytic human leukocyte antigen DR
PCO_2_: Partial pressure of carbon dioxide
PO_2_: Oxygen partial pressure
RR: Respiratory rate
TEAS: Transcutaneous electrical acupoint stimulation
Treg: Regulatory T cell
VT: Tidal volume
